# Mulberry leaf ameliorate STZ induced diabetic rat by regulating hepatic glycometabolism and fatty acid β-oxidation

**DOI:** 10.3389/fphar.2024.1428604

**Published:** 2024-11-20

**Authors:** Bohan Lv, Siyuan Liu, Yaqi Li, Zhigang Li, Yongcheng An, Changhao He, Huilin Zhang, Yan Huang, Wanxin Fu, Quantao Ma, Baosheng Zhao

**Affiliations:** ^1^ Department of Endocrinology, Guanganmen Hospital, China Academy of Chinese Medical Sciences, Beijing, China; ^2^ School of Acupuncture-Moxibustion and Tuina, Beijing University of Chinese Medicine, Beijing, China; ^3^ School of Chinese Materia Medica, Beijing University of Chinese Medicine, Beijing, China; ^4^ School of Life Sciences, Beijing University of Chinese Medicine, Beijing, China; ^5^ Scientific Research Institute of Beijing Tongrentang Co., Ltd., Beijing, China; ^6^ Beijing Research Institute of Chinese Medicine, Beijing University of Chinese Medicine, Beijing, China

**Keywords:** type 2 diabetes, mulberry leaf, proteomics, metabolomics, liver metabolism

## Abstract

**Introduction:**

Type 2 diabetes (T2D) is a metabolic disorder marked by disruptions in glucolipid metabolism, with numerous signaling pathways contributing to its progression. The liver, as the hub of glycolipid metabolism, plays a pivotal role in this context. Mulberry leaf (ML), a staple in traditional Chinese medicine, is widely utilized in the clinical management of T2D. Synthesizing existing literature with the outcomes of prior research, it has become evident that ML enhances glucose metabolism via multiple pathways.

**Methods:**

In our study, we induced T2D in rats through a regimen of high-sugar and high-fat diet supplementation, coupled with intraperitoneal injections of streptozotocin. We subsequently administered the aqueous extract of ML to these rats and assessed its efficacy using fasting blood glucose levels and other diagnostic indicators. Further, we conducted a comprehensive analysis of the rats’ liver tissues using metabolomics and proteomics to gain insights into the underlying mechanisms.

**Results:**

Our findings indicate that ML not only significantly alleviated the symptoms in T2D rats but also demonstrated the capacity to lower blood glucose levels. This was achieved by modulating the glucose-lipid metabolism and amino-terminal pathways within the liver. ACSL5, Dlat, Pdhb, G6pc, Mdh2, Cs, and other key enzymes in metabolic pathways regulated by ML may be the core targets of ML treatment for T2D.

**Discussion:**

Mulberry leaf ameliorate STZ induced diabetic rat by regulating hepatic glycometabolism and fatty acid β-oxidation.

## 1 Introduction

In recent years, the incidence of type 2 diabetes (T2D) has escalated alongside rapid economic development and the prevalence of unhealthy lifestyle and dietary habits. In 2021, it was estimated that approximately 537 million adults globally were living with diabetes, with the majority having T2D. This figure is projected to surge to 784 million by 2045 (Ogurtsova et al., 2022). The underlying pathology of T2D is marked by insulin resistance, hyperinsulinemia, and dysfunction of pancreatic beta-cells (Schwartz et al., 2016). Recent research has highlighted the significant role of factors beyond skeletal muscle, liver, and pancreatic β-cells in T2D progression. These include intestinal insulin dysregulation, lipolysis, persistent hyperinsulinemia, renal glucose reabsorption, and disorders of central appetite regulation (DeFronzo, 2009).

Patients with T2D often experience disruptions in glucolipid metabolism, emphasizing the critical role that maintaining glucose homeostasis plays in both the prevention and treatment of the disease (Fernández-Real et al., 2015). Beyond neuromodulation and hormonal regulation, the liver is a vital participant in this metabolic balance (Radziuk and Pye, 2001). As a key target organ for insulin, the liver is tasked with regulating essential physiological processes, including glycogen synthesis and breakdown, glycolysis, and gluconeogenesis. The capacity for hepatic gluconeogenesis is particularly pivotal, as it directly influences fasting blood glucose levels (Lin et al., 2018), thereby placing the liver at the center of glucose metabolism regulation (Wang et al., 2012; Sharabi et al., 2017).

Mulberry leaf (ML), derived from the dried leaves of Morus alba L., a member of the Moraceae family, is a staple in the clinical treatment of T2D. In our previous study, we analyzed the composition of MLE using UPLC-Q-Exactive Orbitrap-MS, 61 compounds were identified from MLE, they contained 16 flavonoids (rutin, quercetin, kaempferol, et al.), 21 organic acids (vanillic acid, protocatechuic acid, et al.), 9 phenolic compounds (eugenol, moracin A, et al.), 3 alkaloids (1-deoxynojirimycin, et al.), 3 coumarins (5,7-dihydroxycoumarin, et al.), and 9 other categories (vanillin, et al.) (Ma et al., 2022). Research has identified a spectrum of pharmacological properties for ML, including hypoglycemic (Chen et al., 2023), antiviral (Steinmann et al., 2007), antioxidant (Eruygur and Dural, 2019), and anti-tumor activities (Kumkoon et al., 2023). The hypoglycemic mechanism of ML is believed to involve the inhibition of α-glucosidase activity (Fongsodsri et al., 2022), modulation of fat metabolism (Cheng et al., 2022), inhibiting the expression of fatty acid synthase and increasing the activity of liver antioxidant enzymes SOD and catalase (Ren et al., 2015; Zhang et al., 2019; Zhang et al., 2024; Tsai et al., 2024), and amelioration of inflammation (Tian et al., 2019). However, the extent to which ML exerts its hypoglycemic effects through the regulation of hepatic metabolism remains to be fully elucidated.

Proteomics and metabolomics are cutting-edge technologies that offer a snapshot of an organism’s metabolic state at a given time. These disciplines are capable of tracking changes in metabolites and proteins, providing a real-time reflection of the organism’s regulatory networks and unveiling the intricate processes underlying life regulation. Widely utilized in the life sciences, these tools are particularly valuable for studying pathophysiology. In our experiment, we induced T2D in rats through a combination of a high-sugar, high-fat diet and intraperitoneal injections of streptozotocin. The rats were then treated with aqueous extracts of ML. Utilizing proteomics and metabolomics, we assessed the regulatory impact of ML on the liver of T2D rats, aiming to elucidate the mechanisms by which ML exerts its therapeutic effects in the treatment of T2D.

## 2 Materials and methods

### 2.1 Chemicals and reagents

Mulberry leaf (No.20180120, Beijing Tongrentang Co., Ltd., China), metformin (No. AAT8173, Shanghai Squibb Pharmaceuticals Co., Ltd., China), streptozotocin (No. WXBB6772V, Sigma, United States), Glycerin (No. G0854, Sangon Biotech (Shanghai) Co., Ltd. China), bromophenol blue (No. 161-0404, Sangon Biotech (Shanghai) Co., Ltd. China), sodium dodecyl sulfonate (No. 161-0302, Bio-Rad, United States), iodoacetamide (No. 163-2109, Bio-Rad, United States), dithiothreitol (No. 161-0404, Bio-Rad, United States), Urea (No. 161-0731, Bio-Rad, United States), Tris (hydroxymethyl) aminomethane (No. A6141, Sigma, United States), trifluoroacetic acid (No. T6508, Sigma, United States), ammonium acetate (No. 70221, Sigma, United States), ammonia (No. 1336216, Sigma, United States), KH2PO4 (No. 10017618, Sinopharm Chemical Reagent Co., Ltd., China), KCl (No. 10016318, Sinopharm Chemical Reagent Co., Ltd., China), HCl No. 10011018, Sinopharm Chemical Reagent Co., Ltd., China), carboxylic acid (No. 06450, Fluka, United States), CAN (No. 1499230-935, Merck, United States), TMT10 plex Isobaric Label Reagent (No. 90060-90068, Thermo, United States), Trypsin (No. 317107, Promega, United States).

High-sugar and high-fat feed (feed formula: 20% sucrose, 15% lard, 1.2% cholesterol, 0.2% sodium cholic acid, an appropriate amount of casein, calcium hydrogen phosphate, stone powder.) was purchased from Beijing Huafucang Biotechnology Co., Ltd. The calories in the High-sugar and high-fat feed were 5.24 kcal/g, of which 14.1% were from protein, 60% from fat, and 25.9% from carbohydrates.

Composition of regular diet, protein sources (soybean meal, fish meal), fat sources (vegetable oils), fiber sources (wheat bran), carbohydrates (corn, wheat offal), vitamins (Vitamin A, Vitamin D, Vitamin E, Vitamin B1, Vitamin B2, Vitamin B6, pantothenic acid, etc.), and minerals (calcium phosphate, stone powder, iron, copper, manganese, zinc, etc.). The calories in the regular diet were 3.60 kcal/g, of which 14.1% were from protein, 10% from fat, and 75.9% from carbohydrates.

### 2.2 Equipment and analysis software

Equipment: glucose meter and blood glucose test strips (No. 4002886, Shanghai Johnson Medical Equipment Co. China). Acclaim PepMap 100 C18 upper sample column, EASY column C18 analytical column, Human14 multiple affinity chromatography columns, Easy nLC Chromatography System, Q Exactive Mass Spectrometer, micro ultraviolet photometer, Multiskcan FC enzyme marker, low-temperature high-speed centrifuge (Thermo Fisher Scientific, United States). XBRIDGE column (Waters, United States), vacuum centrifugal concentrator (Eppendorf, German), electrophoresis (GE Healthcare, United States). Homogenizer (MP Biomedicals, United States). Mass Spectrometer (AB SCIEX, United States). Ultra-high pressure liquid chromatograph (Agilent, United States). Ultrasonic crusher (Ningbo Xinzhi Biotechnology Co., China). Constant temperature incubator (Shanghai Jinghong Co., China). Votex oscillators (Shanghai Kite, China). Electronic balance (METLER, United States).

Analysis software: Proteome Discoverer 1.4 (Thermo Fisher Scientific, United States). MASCOT 2.2 (Matrix Science, United Kingdom). Perseus 1.3 (Max Planck Institute of Biochemistry, German). SIMCA-P 14.1 (Umetrics AB, Umea, Sweden). XCMS (AB Sciex, United States).

### 2.3 Establishment and administration of type 2 diabetes rats

Preparation of aqueous extract of ML: ML (10.0 kg) was soaked in deionized water (100 L) for 30 min and extracted at 85°C for 10 h. The extracted liquid was concentrated at 3.75 g/mL, −80°C for storage and reserve.

Establishment of T2D rat model: specific pathogen-free male Sprague-Dawley (SD) male rats, procured from SPF (Beijing) Biotechnology Co., Ltd., were housed and maintained in a designated animal laboratory facility. Upon arrival, the rats were randomly assigned to either a control group or a T2D group. The rats in T2D group were placed on a diet high in sugar and fat, whereas the control group received a standard diet, with both groups being fed for a period of 4 weeks. Prior to the induction of diabetes, the T2D rats were fasted for 12 h. Subsequently, they were administered an intraperitoneal injection of a 1% streptozotocin (STZ) citrate buffer solution (0.1 mmol/L, pH adjusted to 4.2-4.5, and kept at 4°C) at a dosage of 35 mg/kg. The control rats received an equivalent volume of the citrate buffer solution without STZ. Seven days post-injection, fasting blood glucose levels were assessed via tail vein blood sampling. Rats with fasting blood glucose levels of 12 mmol/L or higher were deemed to have a successful T2D model.

Treatment administration in T2D rats: following the establishment of the T2D model, the rats were randomly allocated into five groups based on their fasting blood glucose levels and body weight: a T2D group, and four treatment groups receiving metformin (0.2 g/kg) or mulberry leaf (ML) at doses of 4.0, 2.0, or 1.0 g/kg, respectively, with each group consisting of 15 rats. An intragastric administration regime was implemented, where each rat received a daily dose for a period of 12 consecutive weeks. The control and T2D rats not receiving ML were administered an equivalent volume of distilled water. During the treatment period, the diabetics rats continued to be fed a high-sugar, high-fat diet, the rats in control group received a standard diet.

### 2.4 Proteomics methods

#### 2.4.1 Sample collection and processing

The rats were anesthetized by an intraperitoneal injection of 1% pentobarbital sodium (40 mg/kg) after 12 weeks of administration, blood was collected from the abdominal aorta, and livers were collected and stored in a −80°C refrigerator.

The livers of 9 rats were randomly selected from the control group, T2D group, and ML high dose group, and every three in the group were mixed into one, with a total of 3 samples in each group. Liver samples were added with the appropriate amount of SDT lysate, homogenized and broken by homogenizer, and then sonicated, boiled water bath for 15 min, centrifuged at 14,000 *g* for 40 min, the supernatant was taken and filtered through 0.22 µm filter membrane, and the filtrate was collected. 30 μL of protein solution were mixed with DTT to a final concentration of 100 mM, boiling water bath for 5 min, and cool to room temperature. 200 μL of UA buffer was added to the mix, centrifuged at 14,000 g for 15 min in a 10 kD ultrafiltration centrifuge tube, and the filtrate was discarded. 100 μL of IAA buffer was added, shaken for 1 min, reacted for 30 min at room temperature away from light, and centrifuged at 14,000 g for 15 min. 100 μL of UA buffer was added and centrifuged at 14,000 *g* for 15 min. 100 μL of 100 mM TEAB buffer was added and centrifuged at 14,000 *g* for 15 min. 40 μL of trypsin buffer was added, shaken for 1 min, and placed at 37°C for 16–18 h. Peptides were quantified by centrifugation at 14,000 g for 15 min, and 40 μL of 10-fold diluted 100 mM TEAB buffer was added, and centrifuged at 14,000 g for 15 min to collect filtrate, OD280. 100 μg of each sample peptide was taken separately and labeled according to the instructions of Thermo’s TMT labeling kit. Peptide labeling mix was lyophilized and 100 μg was taken, diluted with 300 μL of 0.1% trifluoroacetic acid, transferred to an XBRIDGE column, centrifuged to collect the fractions, 300 uL of pure water was added, centrifuged to collect the aqueous-phase fractions, and the gradient elution was performed. The sample was lyophilized and reconstituted with 12 μL of 0.1% FA.

#### 2.4.2 HPLC-Q exactive-mass spectrometry analysis

The samples were separated using the HPLC (high performance liquid chromatography) liquid system Easy nLC with buffer solution A as 0.1% formic acid aqueous solution and solution B as 0.1% formic acid acetonitrile aqueous solution (acetonitrile is 84%). The column was equilibrated with 95% of liquid A at a flow rate of 300 nL/min, and the liquid-phase gradient was as follows: from 0 to 80 min, the linear gradient of liquid B was from 0% to 55%; from 80 to 85 min, the linear gradient of liquid B was from 55% to 100%; and from 85 to 90 min, liquid B was maintained at 100%. The samples were separated by chromatography and analyzed by mass spectrometry. The detection mode was positive ion, the scanning range of the parent ion was 300–1,800 m/z, the resolution of the primary mass spectrometry was 70,000 (200 m/z), the Maximum IT of the primary mass spectrometry was 50 ms, and the Dynamic exclusion was 60 s. The mass-to-charge ratios of peptides and peptide fragments were collected according to the following method: 20 fragmentation profiles were collected after each full scan (MS2 scan), MS2 fragmentation was by High Energy Collisional Dissociation (HCD), the Isolation window was 2 m/z, the resolution of the secondary mass spectrometry was 35,000 (200 m/z), the Normalized Collision Energy was 30 eV and Underfill was 0.1%.

#### 2.4.3 Data analysis

Raw data obtained from mass spectrometry analyses were processed for identification and quantification using Mascot 2.2 software in conjunction with Proteome Discoverer 1.4. For functional annotation of the identified proteins, we utilized the Blast2GO program, which systematically executes four steps: sequence comparison using BLAST (Basic Local Alignment Search Tool), extraction of Gene Ontology (GO) entries, primary GO annotation, and subsequent annotation augmentation to refine the functional predictions.

Further enrichment and pathway analysis were conducted with the aid of the Kyoto Encyclopedia of Genes and Genomes (KEGG) database, which facilitated the mapping of our dataset onto known biological pathways. To classify samples and proteins based on their expression profiles, we employed Cluster 3.0 software. This analysis led to the generation of hierarchical clustering heatmaps, which were visualized using Java Treeview software. Additionally, the search for direct and indirect interactions among the proteins of interest was conducted using the STRING database. The data retrieved were employed to construct and analyze protein-protein interaction networks, a task that was accomplished using Cytoscape 3.7.1 software.

### 2.5 Metabolomics methods

#### 2.5.1 Sample processing

The livers of 9 rats were randomly selected from the control group, T2D group, and ML high dose group. To prepare the liver tissue samples for analysis, approximately 100 mg of liver tissue was taken and homogenized with 200 μL of water using an MP homogenizer. The homogenization process involved two cycles at a speed of 6.0 m/s for 20 s each, with three repetitions. Following this, 400 μL of a pre-cooled methanol/acetonitrile solution (1:1, v/v) was added to the homogenate. The mixture was then vortexed and subjected to low-temperature ultrasonication for 30 min, repeated twice. After ultrasonication, the samples were rested at −20°C for 60 min to facilitate protein precipitation.

Subsequently, the samples were centrifuged at 13,000 g for 15 min at 4°C. The supernatant (900 μL per tube) was carefully collected, and the remaining liquid was vacuum-dried to obtain a lyophilized powder. This powder was stored at −80°C until required for analysis. For mass spectrometry analysis, the lyophilized powder was re-dissolved in 100 μL of an aqueous acetonitrile solution (acetonitrile: water, 1:1, v/v). The re-dissolved mixture was vortexed to ensure thorough mixing and then centrifuged at 14,000 g for 15 min at 4°C. The clear supernatant was carefully transferred for subsequent mass spectrometry analysis.

#### 2.5.2 UPLC-triple TOF- mass spectrometry analysis

The column temperature was maintained at 25°C with a flow rate of 0.3 mL per minute. The injection volume for each sample was 2 μL. The mobile phase was composed of two solutions: A, which was a mixture of water with 25 mM ammonium acetate and 25 mM ammonia, and B, which was acetonitrile (CAN). The gradient elution program was meticulously designed as follows: it began with an initial condition of 85% B for the first minute. Over the next 11 min (from 1 to 12 min), the proportion of B was linearly reduced to 65%. This was immediately followed by a sharp decrease from 65% to 40% B over a 0.1-min interval (from 12 to 12.1 min). B was then held at 40% for the subsequent 2.9 min (until 15 min). After this period, B was linearly increased back to 85% over a 0.1-min interval (from 15 to 15.1 min), and this condition was maintained for the remainder of the analysis until 20 min. Throughout the entire analytical process, samples were kept at a temperature of 4°C in the autosampler.

After separation via ultra-performance liquid chromatography (UPLC), the samples were subjected to mass spectrometric analysis using a Triple TOF 6600 mass spectrometer. The electrospray ionization (ESI) source conditions, post-hydrophilic interaction liquid chromatography (HILIC) separation, were optimized as follows: ion source gas 1 and gas 2 were both set to 60 arbitrary units; curtain gas was set to 30 arbitrary units; and the source temperature was maintained at 600°C. The time-of-flight (TOF) MS scan was conducted over a mass-to-charge (m/z) range of 60–1,000 Da, while the product ion scan ranged from 25 to 1,000 Da. The accumulation time for TOF MS scan was set to 0.20 s per spectrum, and for the product ion scan, it was 0.05 s per spectrum.

The secondary mass spectra were obtained in high sensitivity mode using an information-dependent acquisition (IDA) method. The declustering potential was set to ± 60 V, with a collision energy of 35 ± 15 eV. The IDA parameters were configured to exclude isotopes within a mass window of 4 Da and to monitor six candidate ions per cycle.

#### 2.5.3 Data analysis

The initial raw data were transformed into the.mzML format using ProteoWizard software, which facilitated subsequent analysis. The XCMS program was then employed for critical data preprocessing steps, including peak alignment, correction of retention times (RT), and the extraction of peak areas. Identification of metabolite structures was achieved through precise mass number matching, with a tolerance of less than 25 parts per million (ppm), followed by secondary spectral matching against our laboratory’s database.

The extracted data from XCMS were normalized against the total peak area to ensure comparability across samples. We conducted multidimensional statistical analyses, which included an unsupervised principal component analysis (PCA) using SIMCA-P 14.1 software. Additionally, we performed unidimensional statistical analyses such as Student's t-test and Folds of Variation (FC) to assess differential expression. Volcano plots were constructed using R software to visualize these differential expressions.

Further, we utilized Cluster 3.0 software with the Euclidean distance algorithm and average linkage for simultaneous classification along two dimensions: samples and protein expression. This clustering approach allowed us to discern patterns and relationships within the data. To represent these patterns visually, we generated hierarchical clustering heatmaps with Java Tree View software. Finally, the data were subjected to trend clustering analysis and annotated with Kyoto Encyclopedia of Genes and Genomes (KEGG) pathways to identify enriched metabolic routes.

### 2.6 Joint analysis of proteomics and metabolomics results

Differential proteins obtained from proteomics and differential metabolites obtained from metabolomics were simultaneously projected to the KEGG pathway, and combined with the changes of proteins and metabolites in different pathways, the networked mechanism of ML regulating the liver for the treatment of type 2 diabetes mellitus was analyzed.

## 3 Results

### 3.1 Hypoglycemic efficacy of the mulberry leaf on type 2 diabetes rats

Building on our previous research, we have demonstrated that mulberry leaf (ML) significantly enhances serum insulin levels, improves the outcomes of the insulin tolerance test (ITT) and oral glucose tolerance test (OGTT), and positively influences other metabolic indices in rats with T2D (Cai et al., 2016). Our findings indicated that ML effectively regulates glucolipid metabolism, bolsters pancreatic islet function, ameliorates insulin resistance, suppresses the expression of inflammatory markers, and alleviates the symptoms of T2D (Duan et al., 2022; Ma et al., 2022). With these profound effects in mind, we measured fasting blood glucose, body weight, as well as food and water intake in the current study to further assess the impact of ML.

**FIGURE 1:**
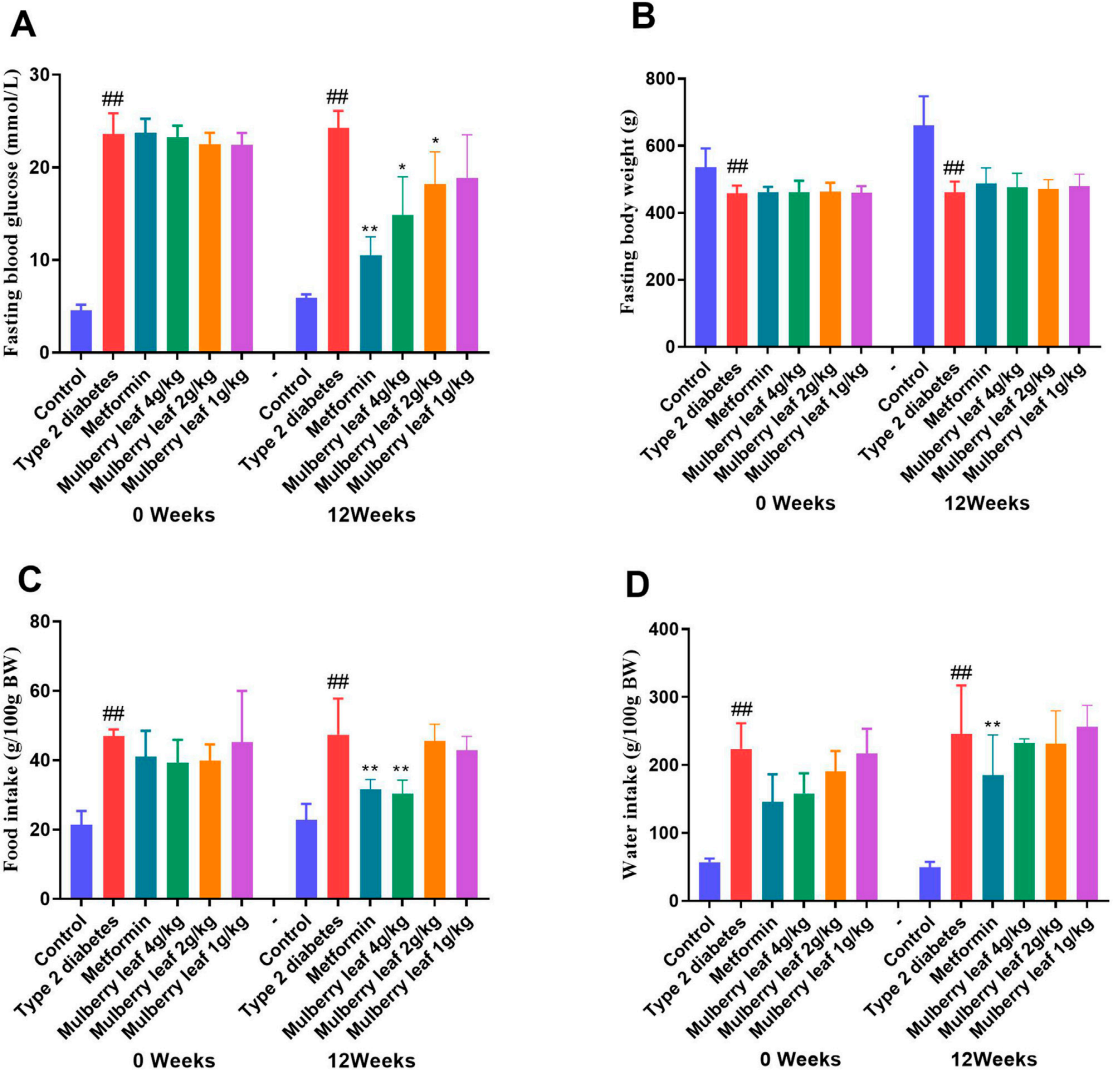
Fasting blood glucose, body weight, food and water intake of rats before and after the ML treatment. **(A)** Fasting blood glucose **(B)** fasting body weight **(C)** food intake, and **(D)** water intake. Data were presented as the mean ± SEM, n = 15. vs. control group, ^#^ *p* < 0.05, ^##^ *p* < 0.01, vs. type 2 diabetes group, *p < 0.05, **p < 0.01.

The research findings indicate that in rats with T2D, fasting blood glucose levels are markedly elevated compared to the control group, along with a significant increase in body weight. Additionally, there is a substantial increase in both food and water consumption, which confirms the successful establishment of the diabetic rat model. The study further demonstrates that treatment with ML results in a notable reduction in fasting blood glucose levels and mitigates the excessive intake of water and food, which are common symptoms in T2D rats. These observations underscore the therapeutic potential of ML in managing T2D, as illustrated in Figure 1 and detailed in Supplementary Tables S1, S2.

### 3.2 Mulberry leaf modulates multiple protein changes in the liver of type 2 diabetes rats

**FIGURE 2:**
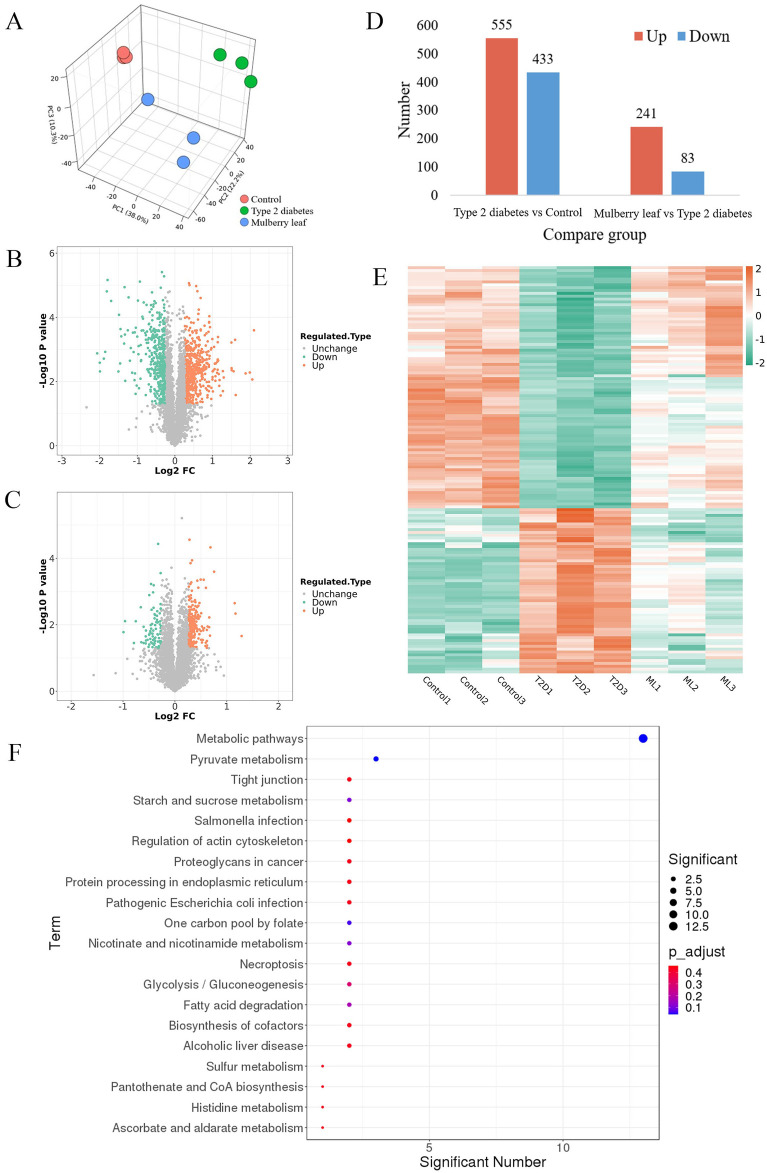
Hepatic proteomics in type 2 diabetes rats treated with mulberry leaf. **(A)** 3D principal component analysis results. **(B)** Volcano plot of protein changes in the liver of type 2 diabetes (T2D) rats. **(C)** Volcano plot of protein changes in the liver of rats treated with mulberry leaf (ML). **(D)** Statistical chart of the number of differential proteins. **(E)** Heat map analysis of core proteins regulated by mulberry leaf. **(F)** Results of KEGG enrichment of core proteins in the liver of type 2 diabetes rats regulated by mulberry leaf.

Proteomics analysis, utilizing principal component analysis (PCA), differentiated the control group, T2D group, and ML treatment group, indicating that ML treatment has a regulatory effect on the liver proteome of T2D rats, as illustrated in Figure 2A. The subsequent volcano plot analysis highlighted significant alterations in several liver proteins of T2D rats, with ML treatment modulating the expression of these proteins, as detailed in Figures 2B, C.

Upon screening for differentially expressed proteins with a fold change magnitude greater than 1.2 and a *p*-value less than 0.05, we identified that 555 proteins were up-regulated and 433 proteins were down-regulated in the liver of T2D rats when compared to the control group. In contrast, in the livers of ML-treated rats, 241 proteins were up-regulated and 83 were down-regulated, as presented in Figure 2D. The intersection of these differential proteins yielded a total of 143 proteins that were reversed by ML treatment in the livers of T2D rats, with 88 proteins up-regulated and 58 down-regulated by ML, as shown in Figure 2E; Supplementary Table S3.

Upon conducting a deeper analysis using the Kyoto Encyclopedia of Genes and Genomes (KEGG) pathway enrichment, it was discovered that the 143 core proteins modulated by ML are integral to several pivotal metabolic pathways. These pathways encompass those associated with glucose metabolism (such as starch and sucrose metabolism, glycolysis, and gluconeogenesis, as well as pyruvate metabolism), lipid metabolism (including fatty acid degradation and the biosynthesis of pantothenate and coenzyme A), and amino acid metabolism (specifically histidine metabolism). This suggests that ML could potentially exert its hypoglycemic effects by modulating a variety of metabolic pathways in the liver of diabetic rats, thereby influencing glucose, lipid, and amino acid metabolism.

Within the metabolic pathways influenced by ML, a select group of differentially regulated proteins serve as critical junctions in these pathways and are likely to be key targets for ML’s therapeutic action on T2D, these include Acsl1, Acox1, Slc25a1, Pdhb, Anxa1, and Apoa1, which are considered potential targets for ML’s intervention. Furthermore, other proteins that interact with these identified targets, such as Fabp2, Ehhadh, Fabp4, Hadh, Echs1, Acaa1a, Pdha1, Dlat, and Mdh2, may also be instrumental in the therapeutic efficacy of ML. Their interplay and significance in the treatment process are visually represented in Figure 2F and elaborated upon in Supplementary Table S3.

### 3.3 Mulberry leaf modulates several metabolites in the liver of type 2 diabetes rats

**FIGURE 3:**
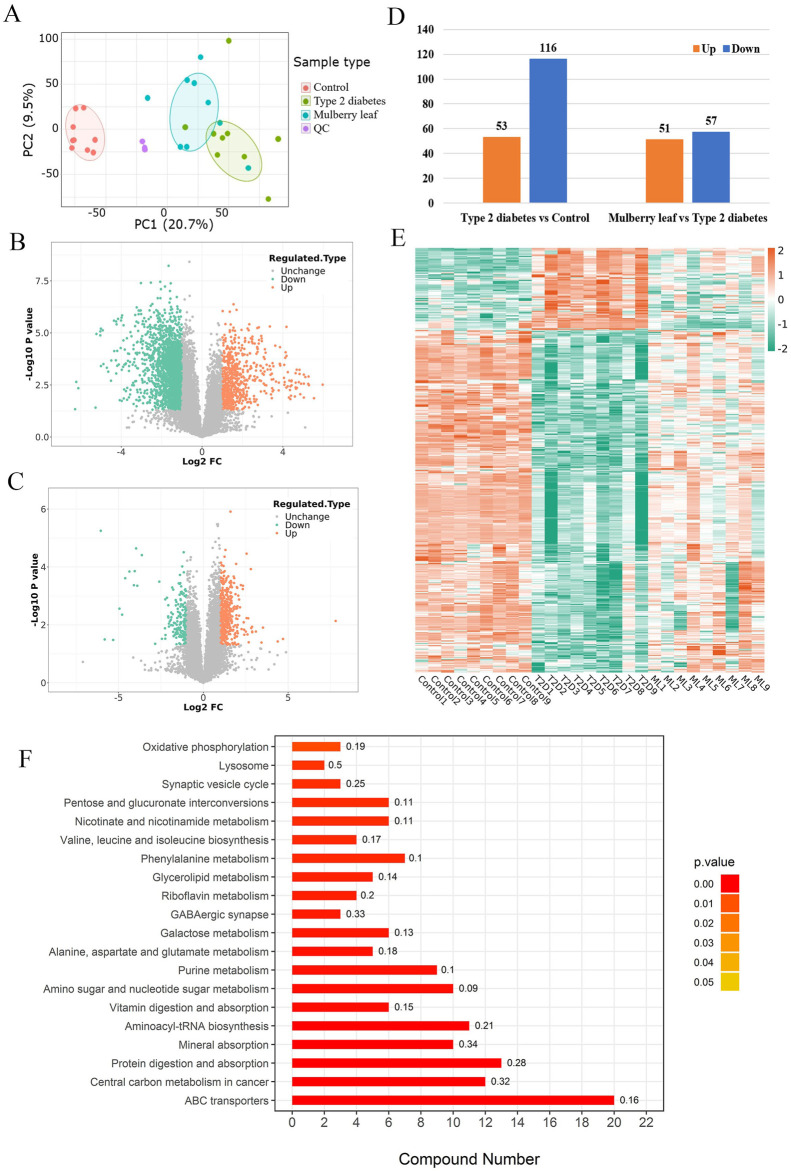
Metabolomics in type 2 diabetes rats treated with mulberry leaf **(A)** Principal component analysis results. **(B)** Volcano plot of metabolites changes in the liver of type 2 diabetes (T2D) rats. **(C)** Volcano plot of metabolite changes in the liver of rats treated with mulberry leaf (ML). **(D)** Statistical chart of the number of differential metabolites. **(E)** Heat map analysis of core metabolites regulated by mulberry leaf. **(F)** Results of KEGG enrichment of core metabolites in the liver of type 2 diabetes rats regulated by mulberry leaf.

The metabolomics analysis, initiated with PCA, successfully distinguished metabolites in the liver of control rats, T2D rats, and those treated with ML. This differentiation indicates that ML exerts a regulatory effect on a spectrum of liver metabolites in T2D rats, as visualized in Figure 3A. The subsequent volcano plot analysis depicted significant regulation of several metabolites within the livers of T2D rats, with ML treatment modulating these metabolites, as detailed in Figures 3B, C.

Upon stringent screening criteria of a fold change magnitude greater than 2 and a *p*-value less than 0.05, we identified substantial alterations in metabolite levels. Specifically, 53 metabolites were up-regulated, while 116 metabolites were down-regulated in the livers of T2D rats when compared to control rats. In contrast, within the ML treatment group, 51 metabolites were upregulated, and 57 were down-regulated, as depicted in Figure 3D. The intersection of these differential metabolites yielded a total of 44 metabolites that were reversed by ML treatment in T2D rats, comprising 15 up-regulated and 29 down-regulated metabolites, as showcased in Figure 3E; Supplementary Table S4.

Furthermore, KEGG pathway enrichment analysis revealed that ML modulates multiple metabolic pathways in the liver of diabetic rats, these pathways include the glucose metabolic pathway (such as pentose and glucuronate interconversions, and galactose metabolism), amino acid metabolic pathways (encompassing valine, leucine and isoleucine biosynthesis, phenylalanine metabolism, and the metabolism of alanine, aspartate, and glutamate), lipid metabolic pathways (like galactose metabolism), and other critical metabolic pathways (including oxidative phosphorylation and lysosomal processes). The analysis suggests that ML may exert its hypoglycemic effects by regulating the interplay of glucose-lipid metabolism, amino acid metabolism, and other metabolic pathways within the liver of diabetic rats. This finding is congruent with the proteomic data, as depicted in Figure 3F, further validating the comprehensive impact of ML on the metabolic landscape in diabetes management.

### 3.4 Analysis of the network mechanism of mulberry leaf modulating hepatic metabolism for the treatment of type 2 diabetes

**FIGURE 4:**
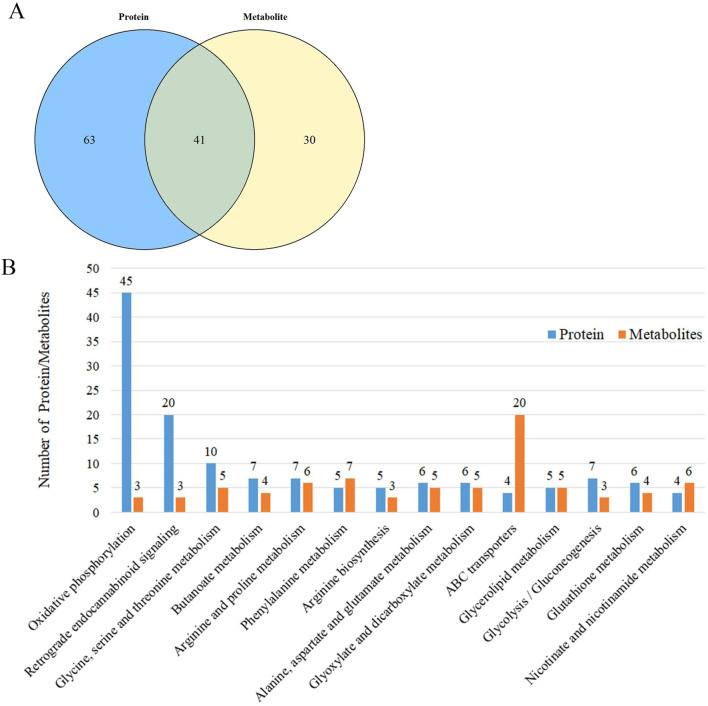
Multi-omics analysis of liver metabolism in type 2 diabetes rats regulated by mulberry leaf **(A)** Enrichment results of differential protein and differential metabolite pathways regulated by mulberry leaf. **(B)** KEGG pathway enrichment results (Number of proteins/metabolites, top 14).

Our comprehensive integrative analysis, which mapped the differential proteins and metabolites regulated by ML onto the KEGG pathways, has pinpointed 41 metabolic pathways that are concurrently influenced by ML, as visualized in Figure 4A and detailed in Supplementary Table S5. By meticulously integrating the count of differentially expressed proteins and metabolites across these pathways, we have distilled 14 pivotal pathways that are subject to ML’s regulatory effects. These key pathways include those involved in glucose metabolism (such as glycolysis/gluconeogenesis), lipid metabolism (encompassing glycerolipid metabolism and glutathione metabolism), amino acid metabolism (covering glycine, serine and threonine metabolism, arginine biosynthesis, and phenylalanine metabolism), and other significant metabolic pathways (like oxidative phosphorylation), as further elucidated in Figure 4B. This refined selection of pathways underscores the multi-targeted therapeutic potential of ML in modulating the complex metabolic dynamics in diabetes.

In our exploration of the network mechanism by which ML exerts its metabolic regulatory effects on the livers of rats with type 2 diabetes (T2D), we have discerned that ML significantly enhances the expression of key enzymes such as ACSL5 and DLAT, this upregulation facilitates the β-oxidation of fatty acids in the liver, a critical process for energy metabolism in T2D rats. Furthermore, ML stimulates the expression of TKFC, which in turn elevates the levels of glycerol phosphate and α-D-glucose within the liver, this increase is instru-mental in propelling the conversion of hepatic glycogen into pyruvate, which subsequently enters the tricarboxylic acid (TCA) cycle, a central pathway for energy production. ML’s influence also extends to the upregulation of Acly, Cs, and Mdh2, enzymes that stimulate the TCA cycle within the liver. Concurrently, ML upregulates the expression of Eno2, Pdha1, Pdhb, and DALT proteins while downregulating G6pc. This coordinated regulation promotes glycolysis and curbs gluconeogenesis in the liver, enhancing the liver’s sugar utilization efficiency. This metabolic reprogramming aids in reducing fasting blood glucose levels, thereby contributing to the amelioration and management of diabetes.

**FIGURE 5:**
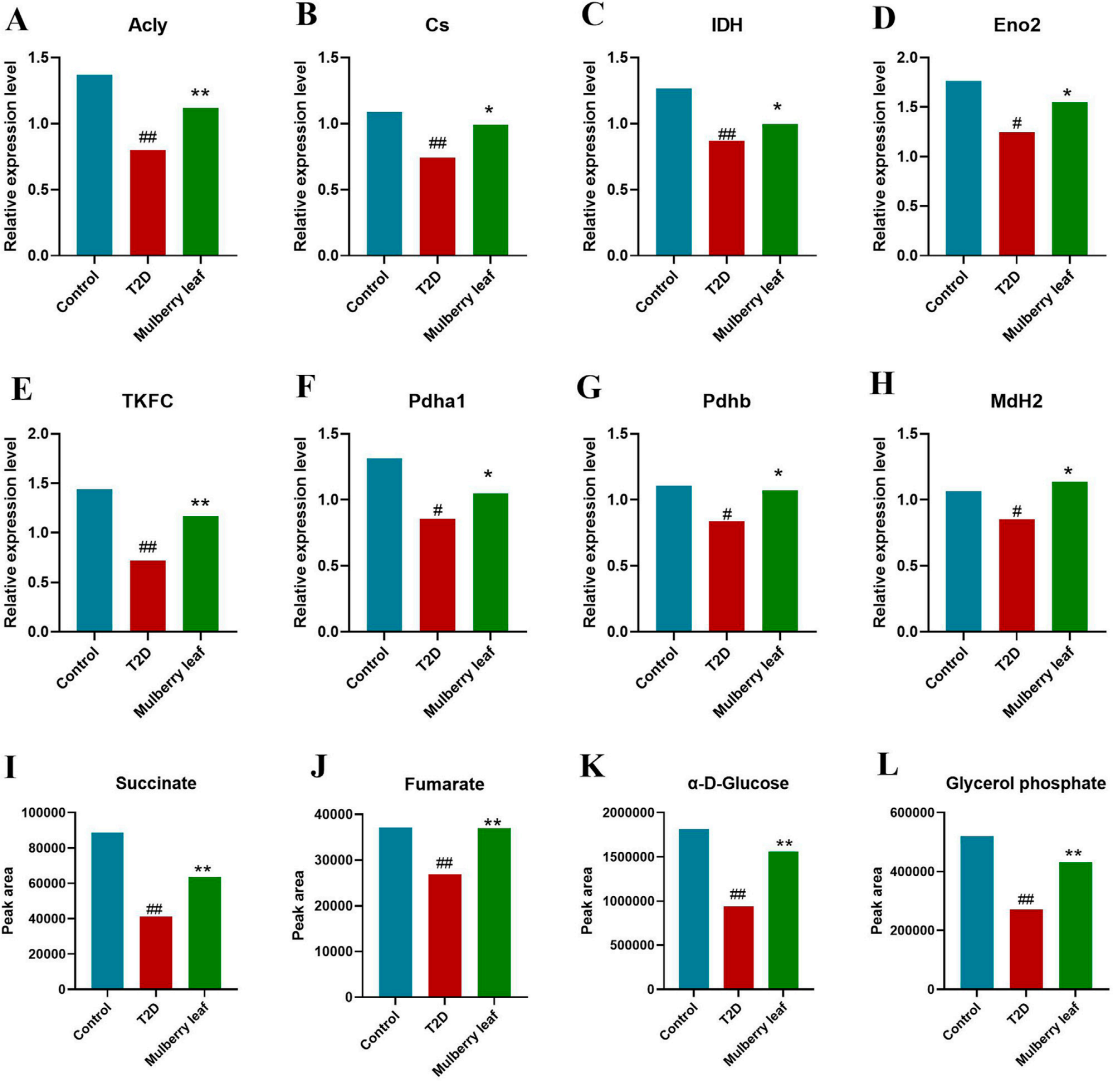
Core differential proteins and metabolites changes in the liver of type 2 diabetes rats with the mulberry leaf treatment **(A)** Acly (ATP citrate lyase). **(B)** Cs (citrate synthase). **(C)** IDH (isocitrate dehydrogenase). **(D)** Eno2 (enolase 2). **(E)** TKFC (triokinase and FMN cyclase). **(F)** Pdha1 (pyruvate dehydrogenase E1 alpha 1 subunit). **(G)** Pdhb (pyruvate dehydrogenase E1 beta subunit). **(H)** MdH2 (malate dehydrogenase 2). **(I)** Succinate. **(J)** Fumarate. **(K)** α-D-Glucose. **(L)** Glycerol phosphate. (vs. control group, ^#^ *p* < 0.05, ^##^ *p* < 0.01, vs. type 2 diabetes group, *p < 0.05, **p < 0.01. mulberry leaf (4 g/kg)).

**FIGURE 6:**
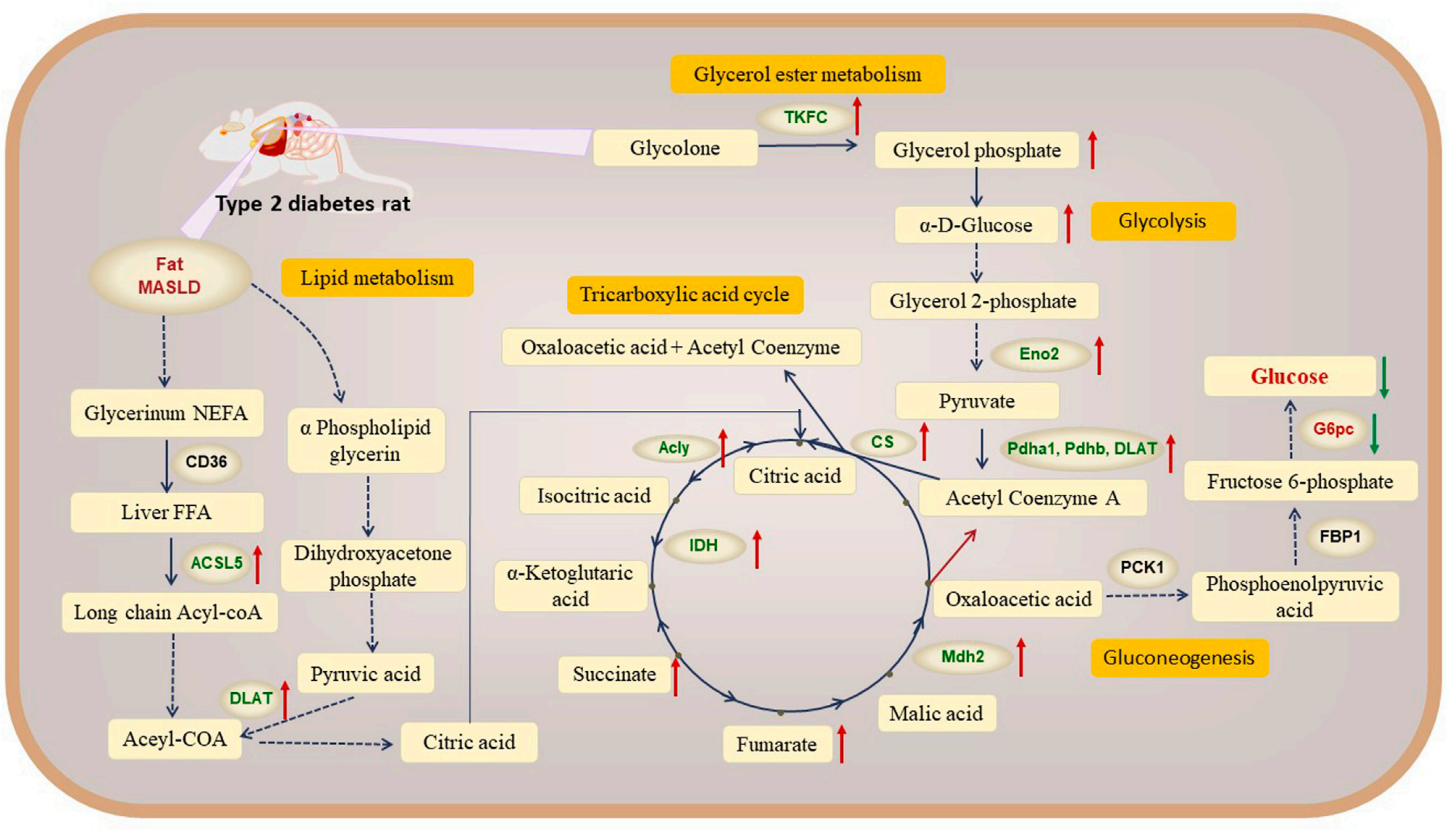
Network mechanism of mulberry leaf in the treatment of type 2 diabetes through modulation of hepatic metabolism (Red indicates metabolic pathways, orange indicates metabolites, blue indicates proteins and red arrows represent mulberry leaf upregulating metabolite or protein levels).

These intricate regulatory actions of ML on the liver’s metabolic machinery are vividly depicted in Figures 5, 6, underscoring its potential as a therapeutic intervention for T2D by modulating a complex array of metabolic pathways and processes.

## 4 Discussion

Type 2 diabetes (T2D) is a common chronic metabolic disorder characterized by multiple metabolic disturbances, it is the third major and frequent disease threatening human health after cancer and cardiovascular and cerebrovascular diseases. The progression of diabetes is intricately linked to disturbances in glucose, lipid, and energy metabolism, with these systems being closely interconnected and mutually influential (Choi, 2022). Maintaining glucose homeostasis in the body is crucial in the prevention and treatment of T2D. The liver, as an important target organ for insulin, regulates physiological processes such as glycogen synthesis and breakdown, glycolysis, and gluconeogenesis. The liver’s gluconeogenic capacity determines the level of fasting blood glucose (Lin et al., 2018), hence the liver plays a key role in the regulation of glucose metabolism (Wang et al., 2012; Sharabi et al., 2017). Impaired glucose tolerance and impaired fasting glycemia are the main clinical manifestations in the early stages of T2D. Once patients progress to T2D, it is difficult for them to return to normal blood glucose levels, necessitating long-term drug therapy, which often comes with side effects. There is an urgent clinical need for the development of highly effective, low-side-effect drugs for diabetes.

Mulberry leave (ML), derived from the dried leaves of the mulberry plant (Morus alba L.) of the Moraceae family, are commonly used in clinical practice as a traditional Chinese medicine for lowering blood glucose. Studies have shown that mulberry leaves can exert hypoglycemic effects by improving inflammation (Duan et al., 2022), enhancing insulin resistance (Tian et al., 2019), and regulating lipid metabolism (Cheng et al., 2022; Cheng et al., 2023). This study used a T2D rat model and evaluated the efficacy of ML treatment for T2D by measuring fasting blood glucose, body weight, and food and water intake. The results showed that ML can significantly reduce the fasting blood glucose levels in T2D rats, improve food and water intake, and have a significant therapeutic effect on T2D. Using proteomic and metabolomic techniques, the study investigated the liver of T2D rats treated with ML, and the results indicated that ML exert their hypoglycemic effects by regulating hepatic glucose metabolism, lipid metabolism, amino acid metabolism, and other metabolic pathways.

### 4.1 Mulberry leave regulates β-oxidation of fatty acids in the liver of T2D rats

In diabetes, the process of aerobic oxidation of glucose is impaired, leading to diminished hepatic glucose utilization. This reduction in glucose-derived energy prompts the organism to rely more heavily on fatty acid oxidation for energy (Holeček, 2023). The substantial increase in fatty acid oxidation, which can overburden the mitochondria, exacerbate oxidative stress, and trigger inflammatory responses and cellular damage. Given that the liver is a central hub for lipid metabolism, dysregulation in this process can significantly contribute to liver insulin resistance (IR). Thus, inhibiting fatty acid oxidation is a crucial strategy in the prevention and control of diabetes (Foley, 1992; Xin et al., 2016).

Our research indicates that T2D rats exhibit signs of obesity and fatty liver disease. ML can significantly upregulate the expression of key proteins in fatty acid β-oxidation, such as ACSL5 and DLAT, promoting the conversion of fatty acids in the liver of T2D rats into acetyl-CoA, Acetyl-CoA combines with oxaloacetic acid to form citric acid, which enters the TCA cycle.

### 4.2 Mulberry leave regulates glycerol ester metabolism, glycolysis and TCA cycle in the liver of T2D rats

Our findings imply that ML has the potential to invigorate the glycerol ester metabolism, glycolysis and the TCA cycle, thereby promoting glucose utilization in the liver. This enhancement in glucose metabolism may represent a significant mechanism by which ML exerts its therapeutic effects in the treatment of diabetic rats.

The research showed that a notable increase in the hepatic content of glycerol phosphate among rats in the ML treatment group. Glycolone is converted to glycerol phosphate by the enzyme Tkfc, we observed an upregulation of Tkfc expression in the livers of ML-treated rats, in contrast to its significant downregulation in the T2D group. Glycerol phosphate, a key molecule that can be further metabolized through glycolysis, along with D-glucose-1-phosphate and α-D-glucose, are pivotal intermediates in this metabolic pathway (Yazdi et al., 2008). Additionally, Enolase 2 (Eno2), a crucial enzyme in glycolysis, facilitates the conversion of 2-phosphoglycerate to phosphoenolpyruvate.

Meanwhile, our study revealed significant up-regulate the expression levels of proteins including malate dehydrogenase 2 (Mdh2), pyruvate dehydrogenase beta (Pdhb), dihydrolipoyllysine acetyltransferase (Dlat), pyruvate dehydrogenase alpha 1 (Pdha1) in the livers of diabetic rats following ML intervention, these are the key catalytic enzymes of the TCA cycle. Pdha1 and Pdhb are responsible for encoding the E1 component of the pyruvate dehydrogenase complex, while DLAT is a critical constituent of this complex. The pyruvate dehydrogenase complex catalyzes the conversion of pyruvate into acetyl-coenzyme A and CO2. Acetyl-coenzyme A, in turn, is a key precursor for the entry of glucose into the tricarboxylic acid (TCA) cycle, also known as the citric acid cycle, for aerobic oxidation. The observed regulation of these metabolites and proteins suggests an increased synthesis of pyruvate and acetyl-coenzyme A in the livers of ML-treated rats, leading to an up-regulation of the citric acid cycle (Bosi et al., 2022; Wang C.-H. et al., 2022).

In summary, ML can upregulate the expression of the Tkfc protein, promoting the conversion of glycolone to glycerol phosphate, glycerol phosphate enters the glycolysis process and is converted to α-D-glucose. ML also upregulate the expression of the Eno2 protein, which promotes the conversion of α-D-glucose into acetyl-CoA, entering the TCA cycle. ML significantly up-regulated the protein expression of Mdh2, Pdhb, Pdha1, Dlat in liver of T2D rats, activated the TCA cycle, and promoted hepatic glucose metabolism.

### 4.3 Mulberry leave regulates gluconeogenesis in the liver of T2D rats

In healthy individuals, blood glucose levels are achieved through a balance between glucose consumption by peripheral tissues and glucose production, with about 90% of glucose production occurring in the liver. Hepatic gluconeogenesis is key to maintaining glucose homeostasis, and targeting hepatic gluconeogenesis for treatment holds significant promise for T2D. In the gluconeogenesis pathway, pyruvate carboxylase (PC), fructose-1,6-bisphosphatase 1 (FBP1), glucose-6-phosphatase (G6pc), and phosphoenolpyruvate carboxykinase (PEPCK) are key enzymes.

In recent years, research on targeting hepatic gluconeogenesis for the treatment of T2D has been emerging. Kfir Sharabi et al. screened active components from small molecule libraries to activate the acetylation of liver peroxisome proliferators-activated receptor γ coactivator α (PGC-1α), inhibit hepatic gluconeogenesis, and improve type 2 diabetes (Sharabi et al., 2017). Research has shown that glucagon stimulates gluconeogenesis through the liver lipolysis mediated by inositol triphosphate receptor 1 (INSP3R1), suggesting that INSP3R1 may be a new target for the treatment of type 2 diabetes (Perry et al., 2020). Yaqiong Chen et al. screened from propolis to target the cAMP response element binding/recombinant CREB regulated transcription coactivator 2 (CREB/CRTC2) transcription complex, regulate hepatic gluconeogenesis, and improve metabolic syndrome in obese mice (Chen et al., 2022). Research indicates that thymocyte selection-associated high-mobility group box factor 4 (TOX4), as a hormone-responsive transcription factor, can act on liver PCK1, inhibition of liver TOX4 can reduce hepatic gluconeogenesis and improve glucose tolerance, suggesting that TOX4 may be a new target for the treatment of diabetes (Wang L. et al., 2022).

In this study, we showed that ML significantly reduced the expression of G6pc, a key protein for gluconeogenesis, inhibited the production of glucose from oxaloacetate via gluconeogenesis, and lowered fasting blood glucose levels in T2D rat.

### 4.4 Mulberry leave regulates amino acid metabolism in the liver of T2D rats

Research has confirmed that amino acid metabolism plays a key role in the occurrence and development of T2D, and amino acids can serve as potential biomarkers for T2D. Branched-chain amino acids (BCAAs), which include valine, leucine, and isoleucine, have been found to be closely related to insulin and diabetes more than 70 years ago (Luetscher, 1942; Felig et al., 1969; Felig et al., 1974). Guaschferré et al. analyzed metabolic studies involving up to 8,000 individuals, including 1,940 T2D patients, and found that increased levels of isoleucine, leucine, and valine lead to an increased relative risk of T2D, while tyrosine and phenylalanine have similar effects. Conversely, glycine and glutamine are negatively correlated with the onset of T2D (Guasch-Ferré et al., 2016). Multiple studies have shown that the levels of valine, leucine, and isoleucine in T2D patients are significantly elevated. Wang et al. followed up with 2,422 non-diabetic individuals over a period of 12 years and found that BCAAs and aromatic amino acids (tyrosine, phenylalanine) in non-diabetic individuals may be important indicators for predicting the onset of diabetes, their research indicates a correlation between BCAAs and diabetes (Wang et al., 2013).

The findings of this study indicate that mulberry leave can significantly downregulate the levels of valine and isoleucine in the liver of rats with T2D. Additionally, the KEGG pathway enrichment results show that ML regulate the metabolic pathways of valine, leucine, and isoleucine, suggesting that ML may improve T2D by downregulating the levels of valine and isoleucine. The study also revealed a significant increase in the level of phenylalanine in the liver of T2D rats, and ML can significantly decrease the phenylalanine levels, suggesting that ML may treat T2D by regulating phenylalanine metabolism.

## 5 Conclusion

ML significantly lowered FBG levels and alleviated the symptoms of T2D in rats. These findings, in conjunction with those from previous studies, suggest that the aqueous extract of ML is an effective therapeutic agent for T2D. In this study, we utilized proteomics and metabolomics to investigate the hepatic regulatory effects of ML on T2D rats. Our results indicate that ML can upregulate the expression of key proteins in fatty acid β-oxidation, such as ACSL5 and DLAT, promoting the β-oxidation of fatty acids in the liver of T2D rats. ML upregulate the expression of the Tkfc protein, promoting the glycerol ester metabolism. ML also upregulate the expression of the Eno2, Mdh2, Pdhb, Pdha1, Dlat, activated the glycolysis and TCA cycle in liver of T2D rats, promoted hepatic glucose metabolism. ML significantly reduced the expression of G6pc, inhibited the gluconeogenesis and lowered fasting blood glucose levels in T2D rat.

Our findings indicate that ML not only significantly alleviated the symptoms in T2D rats but also demonstrated the capacity to lower blood glucose levels. This was achieved by modulating the glucose-lipid metabolism and amino-terminal pathways within the liver. ACSL5, Dlat, Pdhb, G6pc, Mdh2, Cs, and other key enzymes in metabolic pathways regulated by ML may be the core targets of ML treatment for T2D. Further studies are required in a double blind human studies to explore further.

## Data Availability

The proteomics data presented in the study are deposited in the iProX repository, accession number IPX0010035000, the metabolomics data presented in the study are deposited in the Metabolights repository, accession number MTBLS11544.
