# Supplementary material for: Mulberry leaf ameliorate STZ induced diabetic rat by regulating hepatic glycometabolism and fatty acid β-oxidation
Source: Front Pharmacol. 2024 Nov 20;15:1428604. doi: 10.3389/fphar.2024.1428604 (PMC11614592; doi:10.3389/fphar.2024.1428604)
Supplement: Supplementary file 1 [file Table1.DOCX]

**Supplementary Materials**

**Table S1** Fasting blood glucose, body weight of rats before and after the mulberry leaf treatment. (‾*x* ± s)

| Gorup | Dose (g/kg) | N | Fasting blood glucose (mmol/L) | | Fasting body weight（g） | |
| --- | --- | --- | --- | --- | --- | --- |
|  |  |  | 0 week | 12 weeks | 0 week | 12 weeks |
| Control | **--** | 15 | 4.58±0.60 | 5.94±0.36 | 535.82±56.08 | 661.11±86.29 |
| Type 2 diabetes | **--** | 15 | 23.59±2.25^**^ | 24.26±1.85^**^ | 457.58±23.43^**^ | 460.9±32.65^**^ |
| Metformin | 0.2 | 15 | 23.74±1.51 | 10.52±1.98^##^ | 461.71±15.63 | 487.58±46.70 |
| Mulberry leaf | 4.0 | 15 | 23.28±1.23 | 14.88±4.10^#^ | 461.84±33.85 | 475.98±41.73 |
| Mulberry leaf | 2.0 | 15 | 22.5±1.23 | 18.18±3.49^#^ | 463.37±26.65 | 470.31±28.59 |
| Mulberry leaf | 1.0 | 15 | 22.42±1.29 | 18.86±4.65 | 459.56±20.15 | 479.49±36.11 |

Note: vs control, ^*^p<0.05, ^**^p<0.01, vs type 2 diabetes, ^#^p<0.05, ^##^p<0.01.

**Table S2** Food and water intake of rats before and after the mulberry leaf treatment.（‾*x* ± s）

| Gorup | Dose (g/kg) | N | Food intake（g/100g BW） | | Water intake（mL/100g BW） | |
| --- | --- | --- | --- | --- | --- | --- |
|  |  |  | 0 week | 12 weeks | 0 week | 12 weeks |
| Control | **--** | 15 | 21.43±3.98 | 22.76±4.70 | 57.05±5.36 | 49.66±7.70 |
| Type 2 diabetes | **--** | 15 | 47.00±1.91^**^ | 47.41±10.37^**^ | 223.74±37.71^**^ | 245.81±71.30^**^ |
| Metformin | 0.2 | 15 | 40.98±7.52 | 31.53±2.97^##^ | 145.48±41.04 | 184.59±59.80^#^ |
| Mulberry leaf | 4.0 | 15 | 39.36±6.60 | 30.38±3.92^##^ | 158.17±29.84 | 232.03±6.40 |
| Mulberry leaf | 2.0 | 15 | 39.99±4.62 | 45.59±4.84 | 190.18±30.45 | 231.43±48.29 |
| Mulberry leaf | 1.0 | 15 | 45.22±14.83 | 42.89±4.02 | 216.69±36.65 | 256.39±31.45 |

Note: body weight (BW), vs control, ^*^p<0.05, ^**^p<0.01, vs type 2 diabetes, ^#^p<0.05, ^##^p<0.01.

**Table S3** Information of core differential proteins in liver

| NO. | Proteins name | Gene name | T2D VS Control | ML VS T2D |
| --- | --- | --- | --- | --- |
| 1 | fatty acid binding protein 4 | Fabp4 | ↑ ** | ↓ # |
| 2 | androgen-induced 1 | Aig1 | ↑ ** | ↓ ## |
| 3 | AC141959.1 | AC141959.1 | ↑ ** | ↓ # |
| 4 | apolipoprotein A1 | Apoa1 | ↑ ** | ↓ ## |
| 5 | eosinophil peroxidase | Epx | ↑ * | ↓ ## |
| 6 | S100 calcium binding protein A6 | S100a6 | ↑ ** | ↓ # |
| 7 | proliferation and apoptosis adaptor protein 15 | Pea15 | ↑ * | ↓ ## |
| 8 | annexin A1 | Anxa1 | ↑ ** | ↓ # |
| 9 | cytochrome P450, family 2, subfamily c, polypeptide 55-like | LOC100361492 | ↑ ** | ↓ # |
| 10 | enoyl-CoA hydratase and 3-hydroxyacyl CoA dehydrogenase | Ehhadh | ↑ * | ↓ # |
| 11 | fibrillin 1 | Fbn1 | ↑ * | ↓ # |
| 12 | annexin A2 | Anxa2 | ↑ ** | ↓ # |
| 13 | phosphoglycerate dehydrogenase | Phgdh | ↑ ** | ↓ # |
| 14 | cytochrome P450 2B1 | LOC108348266 | ↑ ** | ↓ # |
| 15 | S100 calcium binding protein A10 | S100a10 | ↑ ** | ↓ # |
| 16 | ubiquitin specific peptidase 42 | Usp42 | ↑ * | ↓ # |
| 17 | myosin heavy chain 14 | Myh14 | ↑ ** | ↓ ## |
| 18 | laminin subunit beta 1 | Lamb1 | ↑ * | ↓ # |
| 19 | AHNAK nucleoprotein | Ahnak | ↑ ** | ↓ # |
| 20 | ATP binding cassette subfamily A member 1 | Abca1 | ↑ ** | ↓ # |
| 21 | acyl-CoA oxidase 1 | Acox1 | ↑ ** | ↓ # |
| 22 | minichromosome maintenance complex component 7 | Mcm7 | ↑ ** | ↓ ## |
| 23 | cytochrome P450, family 4, subfamily a, polypeptide 1 | Cyp4a1 | ↑ * | ↓ # |
| 24 | serpin family H member 1 | Serpinh1 | ↑ ** | ↓ # |
| 25 | IFI30, lysosomal thiol reductase | Ifi30 | ↑ ** | ↓ ## |
| 26 | family with sequence similarity 3, member C | Fam3c | ↑ * | ↓ ## |
| 27 | histone cluster 1 H1 family member a | Hist1h1a | ↑ ** | ↓ # |
| 28 | cytoskeleton-associated protein 4 | Ckap4 | ↑ * | ↓ # |
| 29 | clusterin | Clu | ↑ ** | ↓ # |
| 30 | nuclear factor kappa B subunit 2 | Nfkb2 | ↑ ** | ↓ # |
| 31 | fatty acid binding protein 2 | Fabp2 | ↑ ** | ↓ ## |
| 32 | family with sequence similarity 129, member B | Fam129b | ↑ ** | ↓ # |
| 33 | sorting nexin 8 | Snx8 | ↑ ** | ↓ ## |
| 34 | cytoglobin | Cygb | ↑ ** | ↓ ## |
| 35 | S100 calcium-binding protein A4 | S100a4 | ↑ ** | ↓ # |
| 36 | desmin | Des | ↑ ** | ↓ ## |
| 37 | myosin heavy chain 10 | Myh10 | ↑ * | ↓ # |
| 38 | acetyl-CoA acyltransferase 1A | Acaa1a | ↑ * | ↓ # |
| 39 | G protein-coupled receptor 39 | Gpr39 | ↑ ** | ↓ # |
| 40 | glutathione peroxidase 7 | Gpx7 | ↑ ** | ↓ ## |
| 41 | abhydrolase domain containing 6 | Abhd6 | ↑ * | ↓ ## |
| 42 | FGR proto-oncogene, Src family tyrosine kinase | Fgr | ↑ ** | ↓ # |
| 43 | fibulin 1 | Fbln1 | ↑ ** | ↓ ## |
| 44 | neudesin neurotrophic factor | Nenf | ↑ ** | ↓ # |
| 45 | phospholipase D family, member 3 | Pld3 | ↑ ** | ↓ # |
| 46 | cathepsin B | Ctsb | ↑ ** | ↓ # |
| 47 | stomatin | Stom | ↑ * | ↓ # |
| 48 | impact RWD domain protein | Impact | ↑ ** | ↓ ## |
| 49 | tetraspanin 8 | Tspan8 | ↑ ** | ↓ # |
| 50 | filamin A | Flna | ↑ ** | ↓ # |
| 51 | stathmin 1 | Stmn1 | ↑ ** | ↓ # |
| 52 | ezrin | Ezr | ↑ ** | ↓ # |
| 53 | transmembrane protein 120A | Tmem120a | ↑ ** | ↓ # |
| 54 | AC107446.2 | AC107446.2 | ↑ * | ↓ ## |
| 55 | high mobility group nucleosome binding domain 1 | Hmgn1 | ↑ ** | ↓ ## |
| 56 | insulin-like growth factor 2 receptor | Igf2r | ↑ ** | ↓ # |
| 57 | annexin A7 | Anxa7 | ↑ ** | ↓ # |
| 58 | enolase 2 | Eno2 | ↑ ** | ↓ # |
| 59 | ATP citrate lyase | Acly | ↓ ** | ↑ ## |
| 60 | Citrate synthase | Cs | ↓ ** | ↑ # |
| 61 | Isocitrate dehydrogenase | IDH | ↓ ** | ↑ # |
| 62 | arylformamidase | Afmid | ↓ ** | ↓ # |
| 63 | nicotinamide nucleotide adenylyltransferase 3 | Nmnat3 | ↓ * | ↑ # |
| 64 | dihydrolipoamide S-acetyltransferase | Dlat | ↓ ** | ↑ ## |
| 65 | AABR07057436.1 | AABR07057436.1 | ↓ ** | ↑ # |
| 66 | triokinase and FMN cyclase | Tkfc | ↓ ** | ↑ ## |
| 67 | StAR-related lipid transfer domain containing 10 | Stard10 | ↓ ** | ↑ ## |
| 68 | glycogen phosphorylase L | Pygl | ↓ ** | ↑ # |
| 69 | cytochrome b5 reductase 3 | Cyb5r3 | ↓ ** | ↑ ## |
| 70 | alcohol dehydrogenase 6 (class V) | Adh6 | ↓ ** | ↑ # |
| 71 | abhydrolase domain containing 14b | Abhd14b | ↓ ** | ↑ # |
| 72 | glutathione S-transferase alpha 1 | Gsta1 | ↓ ** | ↑ ## |
| 73 | aldo-keto reductase family 1, member C14 | Akr1c14 | ↓ ** | ↑ ## |
| 74 | amylo-alpha-1, 6-glucosidase, 4-alpha-glucanotransferase | Agl | ↓ ** | ↑ # |
| 75 | sideroflexin 1 | Sfxn1 | ↓ ** | ↑ # |
| 76 | neurolysin | Nln | ↓ ** | ↑ ## |
| 77 | pyruvate dehydrogenase E1 alpha 1 subunit | Pdha1 | ↓ ** | ↑ ## |
| 78 | similar to Protein C6orf203 | LOC683897 | ↓ ** | ↑ # |
| 79 | methylmalonyl CoA epimerase | Mcee | ↓ * | ↑ ## |
| 80 | peroxisomal trans-2-enoyl-CoA reductase | Pecr | ↓ ** | ↑ ## |
| 81 | LYR motif containing 4 | Lyrm4 | ↓ ** | ↑ # |
| 82 | glutathione S-transferase alpha 5 | Gsta5 | ↓ ** | ↑ # |
| 83 | prohibitin | Phb | ↓ ** | ↑ # |
| 84 | ATP synthase, H+ transporting, mitochondrial F1 complex, gamma polypeptide 1 | Atp5c1 | ↓ ** | ↑ ## |
| 85 | ketohexokinase | Khk | ↓ ** | ↑ ## |
| 86 | sulfotransferase family 1C member 3 | Sult1c3 | ↓ ** | ↑ # |
| 87 | ATP synthase F1 subunit delta | Atp5f1d | ↓ ** | ↑ # |
| 88 | retinol dehydrogenase 10 | Rdh10 | ↓ ** | ↑ ## |
| 89 | arginase 1 | Arg1 | ↓ ** | ↑ # |
| 90 | reactive intermediate imine deaminase A homolog | Rida | ↓ ** | ↑ ## |
| 91 | sorting nexin family member 30 | Snx30 | ↓ ** | ↑ # |
| 92 | solute carrier family 25 member 1 | Slc25a1 | ↓ ** | ↑ ## |
| 93 | H2A histone family, member J | H2afj | ↓ ** | ↑ # |
| 94 | aldehyde dehydrogenase 1 family, member L2 | Aldh1l2 | ↓ ** | ↑ ## |
| 95 | aldehyde dehydrogenase 1 family, member L1 | Aldh1l1 | ↓ ** | ↑ # |
| 96 | 1,4-alpha-glucan-branching enzyme | Gbe1 | ↓ ** | ↑ # |
| 97 | 1,4-alpha-glucan-branching enzyme | Gbe1 | ↓ ** | ↑ # |
| 98 | ATP synthase F1 subunit alpha | Atp5f1a | ↓ ** | ↑ # |
| 99 | 3-ketodihydrosphingosine reductase | Kdsr | ↓ ** | ↑ ## |
| 100 | aminoadipate aminotransferase | Aadat | ↓ ** | ↑ # |
| 101 | acyl-CoA synthetase medium-chain family member 2 | Acsm2 | ↓ ** | ↑ # |
| 102 | glycerate kinase | Glyctk | ↓ ** | ↑ # |
| 103 | acyl-CoA synthetase long-chain family member 1 | Acsl1 | ↓ ** | ↑ ## |
| 104 | ATP synthase, H+ transporting, mitochondrial Fo complex, subunit d | Atp5h | ↓ ** | ↑ ## |
| 105 | carboxylesterase 1F | Ces1f | ↓ ** | ↑ # |
| 106 | regucalcin | Rgn | ↓ ** | ↑ ## |
| 107 | AC098008.1 | AC098008.1 | ↓ ** | ↑ ## |
| 108 | pyruvate dehydrogenase E1 beta subunit | Pdhb | ↓ ** | ↑ ## |
| 109 | hydroxyacyl-CoA dehydrogenase | Hadh | ↓ ** | ↑ # |
| 110 | potassium calcium-activated channel subfamily N member 4 | Kcnn4 | ↓ ** | ↑ ## |
| 111 | haloacid dehalogenase-like hydrolase domain containing 3 | Hdhd3 | ↓ ** | ↑ # |
| 112 | aldehyde dehydrogenase 2 family (mitochondrial) | Aldh2 | ↓ ** | ↑ ## |
| 113 | taste 1 receptor member 2 | Tas1r2 | ↓ ** | ↑ ## |
| 114 | glycine-N-acyltransferase-like 1 | Glyatl1 | ↓ ** | ↑ ## |
| 115 | aquaporin 4 | Aqp4 | ↓ ** | ↑ # |
| 116 | glutathione peroxidase 1 | Gpx1 | ↓ ** | ↑ ## |
| 117 | nicotinamide phosphoribosyltransferase | Nampt | ↓ ** | ↑ ## |
| 118 | electron transfer flavoprotein regulatory factor 1 | Etfrf1 | ↓ * | ↑ ## |
| 119 | mitochondrial carrier 2 | Mtch2 | ↓ ** | ↑ ## |
| 120 | nudix hydrolase 6 | Nudt6 | ↓ ** | ↑ # |
| 121 | enoyl-CoA hydratase, short chain 1 | Echs1 | ↓ ** | ↑ # |
| 122 | fumarylacetoacetate hydrolase domain containing 1 | Fahd1 | ↓ ** | ↑ ## |
| 123 | malate dehydrogenase 2 | Mdh2 | ↓ ** | ↑ ## |
| 124 | asialoglycoprotein receptor 2 | Asgr2 | ↓ ** | ↑ ## |
| 125 | ATP synthase membrane subunit f | Atp5mf | ↓ ** | ↑ ## |
| 126 | solute carrier family 25 member 13 | Slc25a13 | ↓ ** | ↑ ## |
| 127 | dimethylglycine dehydrogenase | Dmgdh | ↓ ** | ↑ ## |
| 128 | family with sequence similarity 210, member B | Fam210b | ↓ ** | ↑ ## |
| 129 | thiosulfate sulfurtransferase | Tst | ↓ ** | ↑ ## |
| 130 | asialoglycoprotein receptor 1 | Asgr1 | ↓ ** | ↑ ## |
| 131 | ornithine carbamoyltransferase | Otc | ↓ ** | ↑ # |
| 132 | glycine cleavage system protein H | Gcsh | ↓ ** | ↑ ## |
| 133 | serine (or cysteine) proteinase inhibitor, clade A, member 3C | Serpina3c | ↓ ** | ↑ ## |
| 134 | solute carrier family 37 member 4 | Slc37a4 | ↓ ** | ↑ ## |
| 135 | urinary protein 3-like | LOC100912405 | ↓ * | ↑ ## |
| 136 | similar to alpha-2u-globulin | RGD1566134 | ↓ ** | ↑ # |
| 137 | ubiquitin-conjugating enzyme E2D 3 | Ube2d3 | ↓ ** | ↑ ## |
| 138 | malic enzyme 3 | Me3 | ↓ ** | ↑ ## |
| 139 | glutathione S-transferase alpha 2 | Gsta2 | ↓ ** | ↑ # |
| 140 | cysteine sulfinic acid decarboxylase | Csad | ↓ ** | ↑ # |
| 141 | aldehyde oxidase 3 | Aox3 | ↓ ** | ↑ # |
| 142 | amyloid P component, serum | Apcs | ↓ ** | ↑ # |
| 143 | ETHE1, persulfide dioxygenase | Ethe1 | ↓ ** | ↑ ## |
| 144 | Serine protease inhibitor | LOC299282 | ↓ ** | ↑ ## |
| 145 | SOS Ras/Rac guanine nucleotide exchange factor 1 | Sos1 | ↓ ** | ↑ ## |
| 146 | alpha-2u globulin PGCL3 | LOC259244 | ↓ ** | ↑ ## |

Note: type 2 diabetes (T2D), mulberry leaf (ML), "↑" and "↓" represent the increase and decrease of protein content respectively. Compared with control group, *p<0.05, **P<0.01, Compared with T2D group, ^#^p<0.05, ^##^p<0.01.

**Table S4** Information of core differential metabolites in liver

| No. | Metabolites | M/Z | RT (min) | T2DM VS Control | ML VS T2DM |
| --- | --- | --- | --- | --- | --- |
| 1 | Adenosine | 326.11072 | 321.797 | ↓ ** | ↑ # |
| 2 | all cis-(6,9,12)-Linolenic acid | 277.21714 | 85.272 | ↓ ** | ↑ ## |
| 3 | Muramic acid | 310.11437 | 498.539 | ↓ ** | ↑ # |
| 4 | Xylitol | 151.06074 | 465.949 | ↓ ** | ↑ # |
| 5 | Pentadecanoic Acid | 241.21714 | 87.2045 | ↓ ** | ↑ ## |
| 6 | Alpha-D-Glucose | 179.05640 | 570.992 | ↓ ** | ↑ ## |
| 7 | D-Fructose | 239.07757 | 665.338 | ↓ ** | ↑ ## |
| 8 | Dihydroxyacetone phosphate | 229.01185 | 843.398 | ↓ ** | ↑ ## |
| 9 | Ribitol | 211.08260 | 637.962 | ↓ ** | ↑ # |
| 10 | Succinate | 117.01901 | 747.772 | ↓ ** | ↑## |
| 11 | Xanthosine | 285.08373 | 601.050 | ↓ ** | ↑ ## |
| 12 | Equol | 243.10169 | 51.901 | ↓ ** | ↑ ## |
| 13 | Adenosine 3'-monophosphate | 348.07159 | 778.664 | ↓ ** | ↑ # |
| 14 | Acetylcarnitine | 204.12322 | 579.284 | ↓ ** | ↑ ## |
| 15 | Cytosine | 112.05025 | 391.698 | ↓ * | ↑ ## |
| 16 | Deoxycytidine | 228.09823 | 391.607 | ↓ ** | ↑ ## |
| 17 | Inosine | 251.07784 | 242.839 | ↓ ** | ↑ ## |
| 18 | Glutathione disulfide | 613.16058 | 961.375 | ↓ ** | ↑ ## |
| 19 | N-Acetyl-D-glucosamine | 222.09722 | 496.076 | ↓ ** | ↑ ## |
| 20 | (R)-mevalonic acid 5-Phosphate | 229.14379 | 210.001 | ↓ ** | ↑ ## |
| 21 | Folinic acid | 474.17401 | 790.876 | ↓ ** | ↑ ## |
| 22 | Creatinine | 114.06562 | 315.744 | ↓ ** | ↑ # |
| 23 | Nicotinamide adenine dinucleotide (NAD) | 664.11713 | 841.092 | ↓ ** | ↑ ## |
| 24 | L-Carnitine | 162.11257 | 762.711 | ↓ ** | ↑ # |
| 25 | Hypoxanthine | 137.04553 | 413.829 | ↓ ** | ↑ ## |
| 26 | Thymidine | 485.18797 | 185.438 | ↓ ** | ↑ ## |
| 27 | Nicotinamide | 123.05512 | 209.431 | ↓ * | ↑ # |
| 28 | Fumarate | 115.00345 | 721.402 | ↓ ** | ↑ ## |
| 29 | Glycerol phosphate | 152.99550 | 527.368 | ↓ ** | ↑ ## |
| 30 | Valeric acid | 101.06022 | 179.023 | ↑ ** | ↓ ## |
| 31 | alpha-D-Galactose 1-phosphate | 241.01206 | 771.587 | ↑ ** | ↓ ## |
| 32 | Glyceric acid | 105.01870 | 581.485 | ↑ * | ↓ ## |
| 33 | D-Mannose-6-phosphate | 261.03732 | 941.968 | ↑ ** | ↓ ## |
| 34 | D-Proline | 116.07038 | 590.862 | ↑ ** | ↓ # |
| 35 | alpha-D-Glucose 1-phosphate | 243.02683 | 775.450 | ↑ ** | ↓ ## |
| 36 | L-Pyroglutamic acid | 130.04948 | 577.110 | ↑ ** | ↓ # |
| 37 | L-Asparagine | 133.06052 | 715.454 | ↑ ** | ↓ ## |
| 38 | Trans-4-Hydroxy-L-proline | 114.05374 | 527.478 | ↑ ** | ↓ # |
| 39 | Cytidine monophosphate N-acetylneuraminic acid | 615.15540 | 858.424 | ↑ ** | ↓ ## |
| 40 | L-Cystine | 241.03142 | 826.856 | ↑ ** | ↓ ## |
| 41 | Taurine | 126.02174 | 562.190 | ↑ ** | ↓ ## |
| 42 | gamma-L-Glutamyl-L-glutamic acid | 277.10347 | 892.812 | ↑ ** | ↓ ## |
| 43 | Pantothenate | 220.11849 | 530.481 | ↑ ** | ↓ ## |
| 44 | Trimethylamine N-oxide | 76.07535 | 624.466 | ↑ ** | ↓ # |

Note: type 2 diabetes (T2D), mulberry leaf (ML), "↑" and "↓" represent the increase and decrease of metabolite content, respectively. Compared with control group, *p<0.05, **P<0.01, Compared with T2DM group, ^#^p<0.05, ^##^p<0.01. Mass-to-charge ratio (M/Z), retention time (RT).

**Table S5** Information on metabolic pathways regulated by mulberry leave

| NO. | Name of metabolic pathway |
| --- | --- |
| 1 | ABC transporters |
| 2 | Valine, leucine and isoleucine degradation |
| 3 | Glycolysis / Gluconeogenesis |
| 4 | Central carbon metabolism in cancer |
| 5 | Oxidative phosphorylation |
| 6 | Glyoxylate and dicarboxylate metabolism |
| 7 | Pyruvate metabolism |
| 8 | Glycerolipid metabolism |
| 9 | Nicotinate and nicotinamide metabolism |
| 10 | Lysine degradation |
| 11 | Citrate cycle (TCA cycle) |
| 12 | Glycine, serine and threonine metabolism |
| 13 | Glutathione metabolism |
| 14 | Propanoate metabolism |
| 15 | Butanoate metabolism |
| 16 | Cysteine and methionine metabolism |
| 17 | Tyrosine metabolism |
| 18 | Glucagon signaling pathway |
| 19 | Arginine and proline metabolism |
| 20 | Phenylalanine metabolism |
| 21 | Amino sugar and nucleotide sugar metabolism |
| 22 | beta-Alanine metabolism |
| 23 | Drug metabolism - other enzymes |
| 24 | Thyroid hormone synthesis |
| 25 | Fructose and mannose metabolism |
| 26 | Sulfur metabolism |
| 27 | Vitamin digestion and absorption |
| 28 | Pathways in cancer |
| 29 | Pentose phosphate pathway |
| 30 | Ascorbate and aldarate metabolism |
| 31 | Arginine biosynthesis |
| 32 | Starch and sucrose metabolism |
| 33 | One carbon pool by folate |
| 34 | Biosynthesis of unsaturated fatty acids |
| 35 | Lysosome |
| 36 | Glycerophospholipid metabolism |
| 37 | Fatty acid biosynthesis |
| 38 | Histidine metabolism |
| 39 | Taurine and hypotaurine metabolism |
| 40 | cAMP signaling pathway |
| 41 | Insulin resistance |
